# Prevalence, Risk Factors, and Outcomes of Platelet Transfusion Refractoriness in Critically Ill Patients: A Retrospective Cohort Study

**DOI:** 10.1155/2021/5589768

**Published:** 2021-09-23

**Authors:** Saeed Arabi, Abdullah O. Almahayni, Abdulrahman A. Alomair, Emad M. Masuadi, Moussab Damlaj, Hasan M. Al-Dorzi

**Affiliations:** ^1^College of Medicine, King Saud bin Abdulaziz University for Health Sciences, Riyadh, Saudi Arabia; ^2^King Abdullah International Medical Research Center, Riyadh, Saudi Arabia; ^3^Department of Oncology, King Abdulaziz Medical City-Riyadh, Ministry of National Guard Health Affairs, Riyadh, Saudi Arabia; ^4^Intensive Care Department, King Abdulaziz Medical City-Riyadh, Ministry of National Guard Health Affairs, Riyadh, Saudi Arabia

## Abstract

**Background:**

Refractoriness to platelet transfusion is an understudied phenomenon in critically ill patients. Our objective was to evaluate the prevalence, risk factors, and clinical outcomes of platelet refractoriness among patients in a tertiary-care intensive care unit (ICU).

**Methods:**

A retrospective cohort study included all patients (age >14 years) who were admitted to a tertiary-care medical-surgical ICU between 2011 and 2016 and received ≥2 platelet transfusions during their ICU stay. We calculated platelet increment (PI) and corrected count increment (CCI).

**Results:**

A total of 267 patients were enrolled in the study, collectively receiving 1357 transfusions with a median of 4.0 (interquartile range: 2.0, 6.0) transfusions per patient. The median pretransfusion platelet count was 31000.0 × 10^6^/L (interquartile range: 16000.0, 50000.0). The median PI was 6000 × 10^6^/L. The prevalence of platelet transfusion refractoriness was 54.8% based on PI < 10000 × 10^6^/L and 57.0% based on CCI <5000. Patients admitted under hepatology/liver transplant had the highest rates of platelet refractoriness (69.6%), while those under general surgery had the lowest rate (43.2%). Younger age, nontrauma admission, and larger spleen size were associated with platelet refractoriness. Finally, refractoriness was associated with increased length of stay in the ICU (*p* = 0.02), but not with mortality.

**Conclusions:**

Platelet transfusion refractoriness was highly (>50%) prevalent in ICU patients. However, it was not associated with increased mortality.

## 1. Introduction

Thrombocytopenia is commonly seen in critically ill patients. Its incidence and prevalence during intensive care unit (ICU) admission have been reported to be 13–44.1% and 8.3–67.6%, respectively [1]. The variability in epidemiology reflects heterogeneity in patient characteristics and differing thrombocytopenia thresholds [1]. Thrombocytopenia is associated with increased mortality, prolonged ICU and hospital stay, and bleeding, as well as blood product consumption [1, 2]. Prophylactic and therapeutic platelet transfusion is a common practice in the ICU; however, patients may experience platelet transfusion refractoriness, a phenomenon in which the expected posttransfusion platelet count increment is not achieved.

Platelet transfusion refractoriness is predominantly reported in patients with hematologic disorders [3], and its prevalence varies among studies from 4.8 to 49.6% (Table 1) [4–11]. The prevalence was 34% in severe aplastic anemia patients [12], 27.6% in patients receiving multiple platelet transfusions [4], and 10% in patients with acute myeloid leukemia receiving induction chemotherapy [5]. Causes can be immune and nonimmune. Immune-mediated refractoriness is mainly due to alloimmunization to human platelet antigens and human leukocyte antigens (anti-HLA antibodies), which is more common [13, 14]. When associated with HLA alloimmunization, refractoriness causes increased platelet requirements and delayed bleeding [15]. However, anti-HLA antibodies account for less than 30% of total causes of refractoriness [4], and not all patients who get alloimmunized to HLA antigens develop refractoriness [13]. Strategies such as leukocyte reduction, UV-B irradiation, and use of apheresis platelets have reduced the incidence of immune-mediated platelet refractoriness [5], making nonimmune etiologies, such as ABO-incompatibility, transfusion of old platelets, sepsis, disseminated intravascular coagulation (DIC), splenomegaly, bleeding, and medications, underlie most cases of platelet refractoriness [7, 8, 13, 16]. Most of these factors are commonly seen in the ICU.

Irrespective of the underlying etiology, platelet transfusion refractoriness is a clinically important problem in the ICU. It has been associated with increased complications and mortality [8, 13]. However, there is paucity of studies on the prevalence and clinical significance of platelet refractoriness in the ICU setting. Thus, the objectives of this study were to explore the prevalence, risk factors, and clinical outcomes of platelet transfusion refractoriness among patients admitted to the general ICU of a tertiary-care hospital.

## 2. Materials and Methods

### 2.1. Patients and Setting

This was a retrospective cohort study that was conducted in the adult noncardiac ICUs of King Abdulaziz Medical City, Riyadh, Saudi Arabia. The Institutional Review Board of the Ministry of National Guard Health Affairs approved this study. The hospital was a tertiary-care center in Riyadh, with a capacity of >1000 beds treating a variety of medical conditions and specialties including hematology, oncology, and hematopoietic stem cell transplantation. The ICUs collectively had 60 beds servicing medical, surgical, and trauma patients. Multidisciplinary consultant-based teams provided care with in-house on-call physicians 24 hours per day, 7 days per week [17]. Platelets were transfused at the discretion of the treating ICU team as no related protocol existed during the study period. Typically, the prophylactic transfusion threshold was platelet count <10000–20000 × 10^6^/L and therapeutic threshold <50000 × 10^6^/L in the presence of active bleeding or when an invasive procedure was required. In our institution, six units of single donor platelets prepared from whole blood were pooled to produce a single pooled platelet concentrate; apheresis platelets were given upon a specific request from the treating ICU team; otherwise, pooled platelets were given; irradiated platelets were dispatched preferentially to hematology/hematopoietic stem cell transplantation patients where available, and all units of platelets were leukocyte reduced. Each platelet transfusion episode usually consisted of a single concentrate. The time interval between a platelet transfusion episode and the posttransfusion platelet count was determined by the treating ICU team and depended on the clinical condition of the patient.

The study patients included all adults (≥14 years old) admitted to the ICU between 2011 and 2016 and received at least two platelet transfusions during the ICU admission. For patients with more than one ICU admission within the same hospitalization, only the first admission was considered.

### 2.2. Data Collection and Definitions

Data were collected from different sources, primarily the electronic medical records, ICU administrative database, and hospital blood bank database. Collected variables included patient demographics and clinical characteristics on admission (diagnosis, admission category, Glasgow Coma Scale (GCS), and Acute Physiologic Assessment and Chronic Health Evaluation (APACHE) II score). The date, time, platelet product type (pooled or apheresis), irradiation status, and number of units of each transfusion were noted. Platelet count on admission, nadir platelet count during ICU admission, and the platelet counts before and after each transfusion were recorded. When no platelet count could be found in between two or more platelet transfusions, those transfusions were added up and considered as one. When available, we measured the spleen size of each patient within 30 days of admission based on imaging studies in the hospital Picture Archiving and Communication System. Assessed outcomes were platelet transfusion refractoriness, the interval to next platelet transfusion, ICU and hospital length of stay, ICU and hospital mortality, duration of mechanical ventilation, and new tracheostomy insertion.

In this study, we defined refractoriness to platelet transfusions as a platelet increment (PI) of <10000 × 10^6^/L on at least two consecutive occasions within the same ICU admission. PI was calculated by subtracting the pretransfusion from the posttransfusion platelet count. We also used a definition based on the corrected count increment (CCI) for comparison [3]. CCI adjusts the PI for the amount of platelets transfused and for body surface area and is calculated using the following formula [8]:

(1) $$\begin{array}{c} \text{CCI}=\frac{\text{platelet increment}\times \text{body surface area}}{\text{absolute number of transfused platelets}}. \end{array}$$

The absolute number of platelets was estimated at 3.0 × 10^11^ for each unit of apheresis platelets and 3.3 × 10^11^ for each pooled platelet concentrate [18]. Body surface area was calculated using the Mosteller formula as follows [19]:

(2) $$\begin{array}{c} \text{Body surface area}=\sqrt{\frac{\text{weight}\left(\text{kg}\right)\times \text{height}\left(\text{cm}\right)}{3600}}. \end{array}$$

Patients with CCI <5000 on at least two consecutive occasions were considered refractory to platelet transfusion [5, 9, 10]. Splenomegaly was defined as a splenic craniocaudal diameter of >13 cm on an abdominal CT scan or an abdominal ultrasound [20]. Thrombocytopenia was defined as platelet count <150000 × 10^6^/L [21]. DIC was recorded as a likelihood score adapted from the sepsis-induced coagulopathy score, where a higher score indicated a higher likelihood of DIC [22].

### 2.3. Statistical Analysis

Platelet transfusion refractoriness was analyzed using a “wide” data format, where each row represented a different patient and each different data variable was put in a separate column. The PI and CCI for each transfusion episode, time interval to posttransfusion platelet count, and time interval to the next transfusion, which represented time points per patient, were analyzed using a “long” data format, where each row represented a single transfusion and variables that do not change across time had the same value in all the rows. The patient cohort was stratified according to platelet transfusion refractoriness. The time interval to the next transfusion was categorized into two groups: group 1: <48 hours and group 2: ≥48 hours. The 48-hour limit was chosen as it would clinically indicate the requirement for earlier platelet transfusion and because other studies showed that the average time to next transfusion was close to 48 hours [7]. Continuous variables were presented as medians with the first and third quartiles (Q1 and Q3). Categorical variables were presented as frequencies with percentages. The characteristics and outcomes of the different groups were compared using a rank-based nonparametric test (continuous or ordinal data) or the chi-square test (categorical data), as appropriate. The diagnosis of platelet transfusion refractoriness by PI was compared against that by CCI using kappa statistics, sensitivity, and specificity. The relationship between the time interval to the next transfusion and each of PI and CCI was assessed using Spearman correlation (skewed distribution of data).

A binary logistic regression model was used to identify predictors of platelet transfusion refractoriness. Variables with *p* values < 0.25 on univariate analysis were entered in the model [23] in addition to clinically relevant variables. The independent variables entered in the model were age, APACHE II score, admission category, hematologic malignancy, chronic liver disease, sepsis, shock, the first pretransfusion platelet count, spleen size, DIC likelihood score, and the platelet product type (apheresis, apheresis-irradiated, pooled, pooled-irradiated, and mixed) on the first and second transfusion episodes. The results were reported as odds ratio (OR) and 95% confidence interval (CI). Data were analyzed using SPSS v 25. *p* values < 0.05 were considered statistically significant.

## 3. Results

### 3.1. Patients and Transfusion Characteristics

Between 2011 and 2016, 259 patients required platelet transfusion on ≥2 occasions and were included in the study, collectively receiving 1357 platelet transfusions. The characteristics of the study patients are summarized in Table 2. The median age was 58 years (Q1, Q3: 43.5, 70.0), most (57.5%) patients were males, and 84.9% were admitted for a medical reason. About a third (35.5%) had cancer, almost two-thirds (63.3%) were in shock on ICU admission, 59.8% were septic, and 20.1% had a form of bleeding.

The majority (87.6%) of the patients had thrombocytopenia (platelet count <150000 × 10^6^/L) on admission with count <50000 × 10^6^/L present in 45.7%. The rest (12.4%) developed thrombocytopenia during their ICU stay. The median platelet count on admission was 53500.0 × 10^6^/L (Q1, Q3: 28000.0, 98800.0). The median platelet nadir was 18000.0 × 10^6^/L (Q1, Q3: 10000.0, 29000.0). Each patient received platelet transfusion on a median of 4.0 (Q1, Q3: 2.0, 6.0) occasions. The median number of platelet concentrates per each transfusion episode was 1.0 (Q1, Q3: 1.0, 2.0). Most of the transfused platelets were pooled unirradiated (55.3%), while pooled-irradiated platelets accounted for 13.4%, apheresis platelets accounted for 10.2%, and apheresis-irradiated platelets accounted for 14.4%.

The median time interval to next transfusion was 1.0 day (Q1, Q3: 1.0, 2.0). The posttransfusion platelet count was taken a median of 5.0 hours (Q1, Q3: 2.9, 7.6; range: 0, 40.0 hours) after transfusion.

### 3.2. Platelet Increment and Platelet Transfusion Refractoriness

**Figure 1:**
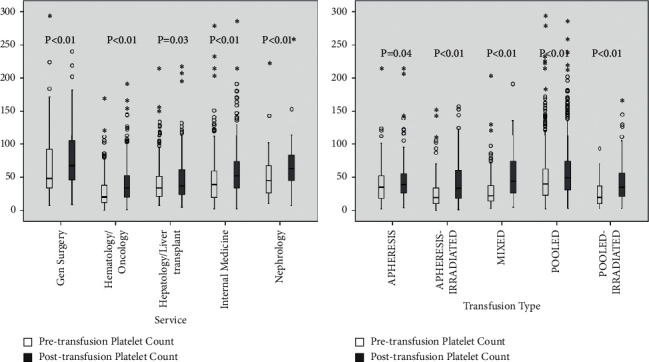
Boxplots of platelet counts before and after platelet transfusion in patient subgroups categorized by the admitting service and by the type of platelet transfused. The platelet increment was statistically different among the different subgroups (*p* = 0.04 among the patients admitted under the different services and *p* < 0.01 among the different platelet product types). Platelet counts on the *Y*-axis are in thousands x10^6^/L.

The median PI was 6000.0 × 10^6^/L (Q1, Q3: −5000.0, 24000.0), and the median CCI was 2800.0 (Q1, Q3: −2000.0, 10800.0).  Figure 1 describes the platelet count before and after platelet transfusion in patient subgroups, categorized by the admitting service and by the transfused platelet product type (apheresis, apheresis-irradiated, pooled, pooled-irradiated, and mixed). The PI was statistically significant in each subgroup and among them. The median PI was the lowest in patients admitted under hepatology/liver transplant (1500.0 × 10^6^/L) followed by those under hematology/oncology (2000.0 × 10^6^/L). The median PI was 6500 × 10^6^/L for patients under general surgery and 0.0 × 10^6^/L for those under internal medicine. The median PI was 3000.0 × 10^6^/L for apheresis platelets, 1000.0 × 10^6^/L for apheresis-irradiated platelets, 11000.0 for pooled platelets, 5000.0 × 10^6^/L for pooled-irradiated platelets, and 11500.0 × 10^6^/L for mixed platelets.

More than half of the patients (54.8%) had platelet transfusion refractoriness by PI (<10000 × 10^6^/L) and 57.0% by CCI (>5000). There was a strong agreement between refractoriness diagnosis by PI and CCI (kappa coefficient: 0.929, 95% CI: 0.884–0.968; *p* < 0.001). Assuming that CCI increment was more accurate for diagnosing platelet refractoriness, diagnosis by PI had 95.2% sensitivity (95% CI: 90.4–98.1%) and 98.2% specificity (95% CI: 93.6–99.8%).

Table 3 shows the prevalence of platelet refractoriness in different patient groups and platelet product types. Trauma patients were the least likely to develop refractoriness (11.1%) compared with medical (56.8%) and nontrauma surgical patients (53.3%). Patients admitted under hepatology/liver transplant had the highest rates of refractoriness (69.6%), while those under general surgery had the lowest rate (43.2%).

**Figure 2:**
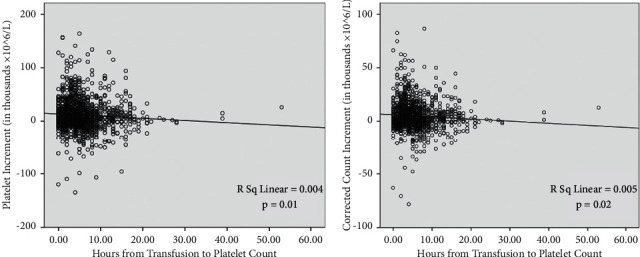
The relationship between the time interval between platelet transfusion and the posttransfusion platelet count and each of the platelet increment (PI) and corrected count increment (CCI). The Spearman r was −0.09 for both PI and CCI.

The relationships between the time interval between transfusion and posttransfusion platelet count and each of PI and CCI are described in Figure 2. The Spearman r was −0.09 for both PI and CCI, indicating no significant linear correlation. As the time interval between platelet transfusion and posttransfusion platelet count was variable, we calculated the prevalence of refractoriness in the different intervals using CCI and PI definition (Table 3). When the analysis was restricted to transfusions which had platelet count measured within 3 hours, the prevalence of platelet refractoriness was 57.4% for CCI <5000 and 63.9% for CCI <7500. When it was restricted to transfusions which had posttransfusion platelet count measured after 12 hours, refractoriness was prevalent in 63.7% for CCI <5000 and 61.1% for CCI <4500.

### 3.3. Predictors of Platelet Transfusion Refractoriness

As shown in Table 2, the admission category, splenomegaly, chronic liver disease, and higher number of platelet transfusions were associated with platelet transfusion refractoriness on univariate analysis. Of patients with splenomegaly, 61.5% developed refractoriness compared to 46.0% of those with nonenlarged spleens (*p* = 0.046). There was a modest, but statistically significant, higher DIC likelihood score among refractory (median score: 5.0, Q1, Q3: 4.0, 6.0) compared to nonrefractory patients (median score: 5.0, Q1, Q3: 4.0, 5.0) (*p* = 0.04).

The multivariable logistic regression model showed that younger age (OR: 0.970 per year increment, 95% CI: 0.952–0.989), nontrauma admission (OR: 11.582, 95% CI: 1.210–110.817), and spleen size (OR: 1.174 per cm increment, 1.053–1.308) were associated with platelet refractoriness. The platelet product type was not associated with refractoriness. The *p* value for the Hosmer and Lemeshow test was 0.20. The area under the curve for the receiver operating characteristic C statistic was 0.728 (95% CI: 0.662–0.794). Both tests indicated that the logistic regression model was a good fit.

### 3.4. Factors Associated with the Time to the Next Platelet Transfusion

Several factors were associated with earlier next transfusion of platelets (Table 4). These included lower pretransfusion platelet count (*p* < 0.001) and lower PI following transfusion (*p* < 0.001). For the relationship with the type of transfused platelet concentrate, retransfusion within 2 days was least frequent with pooled platelets and most common with irradiated-pooled platelets.

### 3.5. Clinical Outcomes

Table 5 describes the clinical outcomes of patients. The median ICU length of stay for all patients was 13.0 days (Q1, Q3: 7.0, 23.0), and the median hospital length of stay was 28.0 days (Q1, Q3: 16.0, 58.0). ICU and hospital mortality were both high, at 59.1% and 73%, respectively.

Compared to nonrefractory patients, those with platelet transfusion refractoriness had a longer stay in the ICU (median of 16.0 days compared to 12.0 days, *p* = 0.015). However, there was no difference in either hospital or ICU mortality. Refractory patients were more likely to have a new tracheostomy tube insertion (*p* = 0.046), but with similar duration of mechanical ventilation (*p* = 0.15).

## 4. Discussion

Much of the published literature on platelet refractoriness has been described in patients with hematologic malignancies or stem cell transplantation. Table 1 summarizes selected important studies on platelet refractoriness [4–11]. In this analysis, we report on the prevalence and clinical outcome of patients with platelet transfusion refractoriness from a large cohort of critically ill patients in a large tertiary-care center.

The reported prevalence of platelet refractoriness ranged from 4.8 to 49.6% with data coming mostly from patients with hematology diseases [4–11]. We observed that >50% of critically ill patients had evidence of platelet transfusion refractoriness. Such value exceeded most of the prior reports on the prevalence of platelet refractoriness in patients with other disorders. This was in spite of the fact that all the platelet products given at our institution were leucocyte reduced, which is well known to reduce the incidence of alloimmunization and, thus, ultimately enhance PI after transfusion [5]. The relatively high prevalence rate of platelet refractoriness in the current study could be related to the inclusion of severely ill patients in an ICU. However, it is possible that the noted prevalence herein was overestimated as the measurement of posttransfusion count was carried out after a median of five hours. However, we did not observe a clear relation between the increment in platelet count and the time interval between transfusion and measurement of posttransfusion platelet count.

CCI is the standard method to measure platelet recovery and survival after transfusion [24, 25]. However, it is cumbersome to use in routine clinical practice; thus, more pragmatic tools such as the PI are routinely utilized. Considering this, we compared these two tools and observed a high concordance. Such information is significant for two reasons; first, the calculation of CCI is frequently based on estimates, not actual counts, of platelet content which is subject to variation. Second, with the emergence of such data showing equivalence among the two methods, clinicians would likely opt to use the more practical PI calculation [26].

Several factors may impact posttransfusion increment in platelet count. In this study, platelet refractoriness was more prevalent in patients admitted under hepatology/liver transplant services. On multivariable logistic regression analysis, we also found that younger age, medical or surgical admission versus trauma, and larger spleen size were associated with higher risk of platelet refractoriness. Other factors that were not studied may be important. These included platelet source and manipulation, ABO matching, and duration of storage in the blood bank [27]. Being a large tertiary-care and trauma center, the platelet storage time in the current study is expected to be short. Furthermore, most platelet products were from pooled platelets, rendering the platelet source and content to be more homogenous.

Refractoriness to platelet transfusions has been associated with adverse clinical outcomes including prolonged hospital stay and increased risk of bleeding as well as mortality (Table 1) [6, 8, 11, 15]. We found that patients with platelet transfusion refractoriness had high mortality, but it was similar to that of patients who did not have refractoriness. Prior data in patients with hematologic malignancies reported that early and late deaths were more common in the refractory group (Table 1), predominantly due to fatal hemorrhage [11]. We speculate that the differences in findings could be due to the increased incidence of immune causes of refractoriness in patients with hematologic malignancies. Such alloimmunization renders the patient more refractory and ultimately at increased risk for severe bleeding episodes.

This analysis carries multiple limitations that emanate mainly from the retrospective single-center study design. The measurement of platelet count after transfusion was carried out at different time intervals. Ideally, it should be carried out within an hour after transfusion to offset any pooling of platelets that subsequently occurs in the spleen. For the calculation of CCI, we used an estimate of platelet content in each of the transfused units and used a CCI cutoff of 5000 to define platelet refractoriness [5, 9, 10]. Other cutoffs have been used such as <7500 at 1 hour and <4500 at 24 hours [8]. Additionally, we did not have data about the immunization of patients against platelet surface antigens. These factors affect the interpretation of the current study and comparing our results with those of others. A number of important points should be highlighted. First, to our knowledge, this is the first analysis of platelet refractoriness in the critical care setting and sheds some insight on its prevalence and outcome in such patients. Second, we used two methods to estimate platelet refractoriness and demonstrated that they were concordant. Nevertheless, this study should be considered as pilot and our findings require further validation in large prospective studies.

## 5. Conclusions

In conclusion, critically ill patients receiving at least two transfusions of platelets had high (>50%) prevalence of platelet transfusion refractoriness, defined by PI < 10000 × 10^6^/L and CCI < 5000. Younger age, nontrauma admissions, and larger spleen size were associated with higher risk of platelet refractoriness. The mortality rate of our patients was high, but platelet transfusion refractoriness was not associated with increased mortality.

## Figures and Tables

**Table 1:** Selected studies evaluating prevalence and outcomes of platelet refractoriness.

Study	Type	Population	Method	Prevalence	Clinical outcomes
Legler et al., 1997 [4]	Prospective cohort	145 patients with hemato-oncological disease at a single center, who received at least two platelet transfusions	Platelet counts repeatedly after ≥two successive platelet transfusions within 48 hours < 20000 × 10^6^/L (16-hr count) or <10000 × 10^6^/L (24-hr count)	27.6%	Not assessed

TRAP study, 1997 [5]	Randomized clinical trial	530 patients receiving induction chemotherapy for acute myeloid leukemia	CCI <5000 after two sequential transfusions	10%	No comparison between refractory and nonrefractory patients reported

Meehan et al., 2000 [6]	Prospective cohort	292 consecutive patients at a single hospital who received platelet transfusions over a six-month period	Three consecutive days of platelet transfusions produced posttransfusion platelet count increments (corrected or uncorrected) ≤ 5000 × 10^6^/L	21.6%	Among refractory patients (compared to nonrefractory)
					(i) Increased mean platelet use (units): 20.6 vs. 2.8, *p* < 0.0001
					(ii) Increased mean hospitalization costs (US $): 103,956 vs. 36,818, *p* < 0.0001
					(iii) Increased mean length of stay: 35.0 vs. 14.4 days, *p* < 0.0001

Slichter et al., 2005 [7]	Ad hoc analysis of a randomized clinical trial	533 patients receiving induction chemotherapy for acute myeloid leukemia	2 sequential 1-hour posttransfusion platelet increments of less than 11000 × 10^6^/L	27%	Not assessed

Kerkhoffs et al., 2008 [8]	Ad hoc analysis of a randomized clinical trial	117 patients hospitalized in the hematology ward who needed or were expected to need 2 or more platelet transfusions	1-hour CCI <7500 and/or a 24-hour CCI <4500	49.6%	Among refractory (compared to nonrefractory patients)
					(i) Decreased 100-day survival (83% vs. 98%, *p* < 0.01)
					(ii) Decreased median survival (491.0 days (Q1, Q3: 156.0, 858.0) vs. 825 days (Q1, Q3: 355.0, 996.0), *p* = 0.032)
					(iii) Increased risk of bleeding: odds ratio: 3.4; 95% CI: 1.1–11

Ferreira et al., 2011 [9]	Prospective cohort	16 adult oncology/hematology patients	Two successive transfusions with CCI <5000	19% (3 patients)	Not assessed

Hess et al., 2016 [10]	Secondary analysis of a randomized clinical trial	734 hematology-oncology patients receiving at least 2 platelet transfusions	Two consecutive CCIs of ≤5000	14%	Not assessed

Comont et al., 2017 [11]	Retrospective cohort	897 adult patients with acute myeloid leukemia receiving intensive chemotherapy	Persistent thrombocytopenia <10000 × 10^6^/L despite at least two successive daily platelet transfusions	4.8%	Among patient with refractoriness (compared to those without)
					(i) Increased severe bleeding events (22% vs. 4.1%, *p* < 0.0001)
					(ii) Increased early death caused by bleeding (12.2% vs. 1.4%, *p* = 0.0006)
					(iii) Increased death by bleeding at any time (24.4% vs. 5.3%, *p* < 0.0001)

CCI: corrected count increment, CI: confidence interval, Q1: first quartile, Q3: third quartile.

**Table 2:** General characteristics of patients by platelet transfusion refractoriness status based on the platelet increment (<10000 × 10^**6**^/L) definition.

A. Patient characteristics		Total (*N* = 259)	Refractory (*N* = 142)	Nonrefractory (*N* = 117)	*p* value
**Age (years)**	Median (Q1, Q3)	58.0 (43.5, 70.0)	57.0 (42.2, 68.0)	60.0 (46.0, 72.0)	0.11
**Male sex**	*N* (%)	149 (57.5)	81 (57.0)	68 (58.1)	0.86
**Body mass index (kg/m** ^**2**^ **)**	Median (Q1, Q3)	27.3 (22.4, 32.3)	27.9 (22.3, 32.9)	26.6 (22.6, 31.4)	0.40
**Obese >30 kg/m**^**2**^	*N* (%)	94 (37.5)	54 (39.4)	40 (35.1)	0.60
**Body surface area (m** ^**2**^ **)**	Median (Q1, Q3)	1.8 (1.6, 1.9)	1.8 (1.7, 1.9)	1.8 (1.6, 1.9)	0.42
**Admission category**	*N* (%)				
Medical		220 (84.9)	125 (88.0)	95 (81.2)	0.03
Surgical		30 (11.6)	16 (11.3)	14 (12.0)	
Trauma		9 (3.5)	1 (0.7)	8 (6.8)	
**Chronic cardiac disease**/238 patients^∗^	*N* (%)	35 (15.2)	18 (13.6)	17 (17.3)	0.44
**Chronic immune disease**/238 patients^∗^	*N* (%)	89 (38.7)	47 (35.6)	42 (42.9)	0.26
**Chronic liver disease**/238 patients^∗^	*N* (%)	48 (20.9)	35 (26.5)	13 (13.3)	0.01
**Chronic respiratory disease**/238 patients^∗^	*N* (%)	17 (7.4)	12 (9.1)	5 (5.1)	0.25
**Chronic renal disease**/238 patients∗	*N* (%)	29 (12.6%)	14 (10.6%)	15 (15.3%)	0.29
**APACHE II score**	Median (Q1, Q3)	25.0 (20.0, 30.0)	25.0 (21.0, 30.0)	24.0 (20.0, 28.5)	0.40
**Glasgow Coma Scale on ICU admission**	Median (Q1, Q3)	14.0 (9.5, 15.0)	14.0 (9.0, 15.0)	14.0 (10.0, 15.0)	0.97
**Mechanical ventilation**/238 patients∗	*N* (%)	191 (83.0)	110 (83.3)	81 (82.7)	0.89
**PaO**_**2**_ **/FiO**_**2**_ **ratio**	Median (Q1, Q3)	178.5 (113.2, 270.5)	176.0 (112.2, 258.0)	182.0 (114.0, 282.5)	0.38
**Shock**	*N* (%)	164 (63.3)	91 (64.1)	73 (62.4%)	0.78
**Sepsis**	*N* (%)	155 (59.8%)	88 (62.0)	67 (57.3)	0.44
**Septic shock**	*N* (%)	149 (57.5%)	84 (59.2)	65 (55.6)	0.56
**DIC likelihood score**	Median (Q1, Q3)	5.0 (4.0, 6.0)	5.0 (4.0, 6.0)	5.0 (4.0, 5.0)	0.04
**Active bleeding**	*N* (%)	52 (20.1)	27 (19.0)	25 (21.4)	0.64
**Active cancer**	*N* (%)	92 (35.5)	47 (33.1)	45 (38.5)	0.37
**Hematological cancer**	*N* (%)	75 (29.0)	38 (26.8)	37 (31.6)	0.39
**Organ transplant**	*N* (%)	13 (5.0)	9 (6.3%)	4 (3.4)	0.28
**Spleen size (cm)**	Median (Q1, Q3)	11.5 (9.4, 14.0)	12.5 (10.5, 14.4)	10.7 (8.9, 13.2)	0.001
**Splenomegaly**/178 patients∗	*N* (%)				0.046
**Present**		65 (36.5)	40 (43.5)	25 (29.1)	
**Absent**		113 (63.5)	52 (56.5)	61 (70.9)	
**Platelet count on ICU admission (x10** ^**6**^ **/L)**	Median (Q1, Q3)	53500.0 (28000.0, 98800.0)	49000.0 (26000.0, 93000.0)	59000.0 (35000.0, 108000.0)	0.11
**Thrombocytopenia on admission (<150000 x 10** ^**6**^ **/L)**	*N* (%)	226 (87.6%)	123 (87.2%)	103 (88.0%)	0.85
**Severe thrombocytopenia on admission (<50000 x 10** ^**6**^ **/L)**	*N* (%)	118 (45.7%)	72 (51.1%)	46 (39.3%)	0.06
**Lowest platelet count during ICU stay (x10** ^**9**^ **/L)**	Median (Q1, Q3)	18.0 (10.0, 29.0)	14.0 (7.0, 28.0)	19.0 (11.0, 34.0)	0.001
**INR**	Median (Q1, Q3)	1.6 (1.3, 2.0)	1.6 (1.3, 2.2)	1.6 (1.2, 2.0)	0.38
**Lactic acid (mmol/L)**	Median (Q1, Q3)	2.9 (1.7, 6.0)	2.9 (1.5, 5.9)	3.0 (1.8, 6.1)	0.47
**Creatinine (*μ*mol/L)**	Median (Q1, Q3)	142.5 (73.8, 256.2)	145.0 (82.2, 260.2)	134.5 (65.8, 232.0)	0.33
**Bilirubin (*μ*mol/L)**	Median (Q1, Q3)	40.0 (19.0, 107.2)	40.0 (19.0, 106.0)	40.0 (19.5, 107.5)	0.99
**Platelet transfusion refractoriness (CCI definition)**/256 patients∗	*N* (%)	146 (57.0%)	139 (98.6%)	7 (6.1%)	<0.001

**B. Transfusion characteristics**		**Total (*N*** **=** **1357)**	**Refractory (*N*** **=** **926)**	**Nonrefractory (*N*** **=** **431)**	*p * **value**
**Platelet product type**	*N* (%)				0.06
Apheresis		138 (10.2%)	98 (10.6%)	40 (9.3%)	
Apheresis-irradiated		196 (14.4%)	124 (13.4%)	72 (16.7%)	
Pooled		750 (55.3%)	508 (54.9%)	242 (56.1%)	
Pooled-irradiated		182 (13.4%)	123 (13.3%)	59 (13.7%)	
Mixed∗∗∗		91 (6.7%)	73 (7.9%)	18 (4.2%)	
**Platelet count prior to transfusion (x10** ^**6**^ **/L)**	Median (Q1, Q3)	31000.0 (17000.0, 52000.0)	31000.0 (16000.0, 52000.0)	33000.0 (18000.0, 54000.0)	0.17
**Number of transfusion episodes per patient**	Median (Q1, Q3)	4.0 (2.0, 6.0)	5.0 (3.0, 8.0)	3.0 (2.0, 4.0)	<0.001
**Number of platelet concentrates per transfusion episode∗∗**	Median (Q1, Q3)	1.0 (1.0, 2.0)	1.0 (1.0, 2.0)	1.0 (1.0, 1.0)	<0.001
**Time to next platelet transfusion (days)**	Median (Q1, Q3)	1.0 (1.0, 2.0)	1.0 (1.0, 2.0)	1.0 (1.0, 2.3)	0.006
**Platelet increment (x10** ^**6**^ **/L)**	Median (Q1, Q3)	6000.0 (−5000.0, 24000.0)	2000 (−8000, 14000)	21000.0 (9000.0, 42000.0)	<0.001
**Corrected count increment (x10** ^**6**^ **/L)**	Median (Q1, Q3)	2800.0 (−2000.0, 10800.0)	600.0 (−3700.0, 5800.0)	10300.0 (3800.0, 18800.0)	<0.001

^∗^For patients with missing data, the denominator is the number of patients with valid observations. For the calculation of percentages, only valid observations are used in the numerator and denominator. ∗∗Six units of single donor platelets prepared from whole blood were pooled to produce a single pooled platelet concentrate.^∗∗∗^Mixed indicates aggregate transfusions that were derived from transfusions with 2 or more different platelet products. APACHE: Acute Physiologic Assessment and Chronic Health Evaluation; CCI: corrected count increment DIC: disseminated intravascular coagulation; ICU: intensive care unit; INR: International Normalized Ratio, Q1: first quartile, Q3: third quartile.

**Table 3:** Prevalence of platelet refractoriness based on platelet increment (PI) and different cutoffs for the corrected count index (CCI), in different patient subgroups and according to the platelet product types and the time interval between platelet transfusion and the posttransfusion platelet count.

	Prevalence of platelet refractoriness	
	PI < 10000 × 10^6^/L	CCI <5000
**All patients** (*N* = 259)	54.8%	57.0%
**Admission category**		
Medical (*N* = 220)	56.8%	58.5%
Surgical (*N* = 30)	53.3%	60%
Trauma (*N* = 9)	11.1%	11.1%
**Admitting service**		
Internal medicine (*N* = 74)	54.5%	56.8%
Hematology/oncology (*N* = 72)	58.9%	56.9%
Hepatology/liver transplant (*N* = 46)	69.6%	71.7%
General surgery (*N* = 37)	43.2%	45.9%
**Splenomegaly^∗^** (*N* = 65)	61.5%	64.6%
**No splenomegaly** (*N* = 113)	46.0%	50%
**Platelet product type**		
Apheresis (*N* = 139^∗∗^)	62.6%	64%
Apheresis-irradiated (*N* = 195^∗∗^)	46.9%	47.7%
Pooled (*N* = 747^∗∗^)	59.1%	61.6%
Pooled-irradiated (*N* = 179^∗∗^)	47.3%	50.3%
Mixed^∗∗∗^ (*N* = 90^∗∗^)	48.4%	65.6%
**Time interval between platelet transfusion and the posttransfusion platelet count**		
<3 hours (*N* = 319^∗∗^)	55.4%	57.4% (63.9% for CCI <7500)
3–6 hours (*N* = 583^∗∗^)	51.3%	55.2%
7–12 hours (*N* = 292^∗∗^)	61.0%	63.4%
>12 hours (*N* = 113^∗∗^)	60.3%	63.7% (61.1% for CCI <4500)

^∗^Spleen size was known in 178 patients. For the calculation of percentages, only valid observations are used in the numerator and denominator. ^∗∗^Transfusion episodes. ^∗∗∗^Mixed indicates aggregate transfusions that were derived from transfusions with 2 or more different platelet products.

**Table 4:** Characteristics of platelet transfusions by the time to the next transfusion.

		Total (*N* = 1118)	Less than 2 days (*N* = 643)	2 days or more (*N* = 475)	*p* value
**Platelet count prior to transfusion (x10** ^**6**^ **/L)**	Median (Q1, Q3)	31000.0 (16000.0, 50000.0)	28000.0 (14000.0, 45000.0)	37000.0 (19000.0, 58000.0)	<0.001
**Number of platelet concentrates per transfusion episode^∗^**	Median (Q1, Q3)	1.0 (1.0, 2.0)	1.0 (1.0, 2.0)	1.0 (1.0, 1.0)	0.04
**Platelet increment (x10** ^**6**^ **/L)**	Median (Q1, Q3)	6000.0 (−5000.0, 24000.0)	5000.0 (−6000.0, 19000.0)	9000.0 (−4000.0, 29000.0)	<0.001
**Platelet product type**	N (%^∗∗^)				
Apheresis		111	71 (64.0)	40 (36.0)	0.16
Apheresis-irradiated		163	101 (62.0)	62 (38.0)	0.23
Pooled		608	310 (51.0)	298 (49.0)	<0.001
Pooled-irradiated		157	109 (69.4)	48 (30.6)	0.001
Mixed^∗∗∗^		79	52 (65.8)	27 (34.2)	0.13
**Time to the next platelet transfusion (days)**	Median (Q1, Q3)	1.0 (1.0, 2.0)	1.0 (1.0, 1.0)	3.0 (2.0, 4.0)	<0.001

^∗^Six units of single donor platelets prepared from whole blood were pooled to produce a single pooled platelet concentrate. ^∗∗^The denominator is the total number of transfusion episodes of the platelet product type. ^∗∗∗^Mixed indicates aggregate transfusions that were derived from transfusions with 2 or more different platelet products. Q1: first quartile, Q3: third quartile.

**Table 5:** Outcomes of patients by platelet refractory status based on the platelet increment (<10000 × 10^6^/L) definition.

		Total (*N* = 259)	Yes (*N* = 142)	No (*N* = 117)	*p* value
**ICU mortality**	*N* (%)	153 (59.1)	86 (60.6)	67 (57.3)	0.55
**Hospital mortality**	*N* (%)	189 (73.0)	105 (73.9)	84 (71.8)	0.70
**New tracheostomy**/238 patients^∗^	*N* (%)	25 (10.9)	19 (14.4)	6 (6.1)	0.046
**Duration of mechanical ventilation (days)**/238 patients^∗^	Median (Q1, Q3)	10.0 (6.0, 17.0)	10.0 (5.0, 15.8)	12.0 (6.0, 19.5)	0.15
**ICU length of stay (days)**	Median (Q1, Q3)	13.0 (7.0, 23.0)	16.0 (8.0, 26.0)	12.0 (6.0, 21.0)	0.02
**Hospital length of stay (days)**	Median (Q1, Q3)	28.0 (16.0, 58.0)	33.0 (16.0, 62.5)	27.0 (14.0, 47.0)	0.11

^∗^For patients with missing data, the denominator is the number of patients with valid observations. For the calculation of percentages, only valid observations are used in the numerator and denominator. ICU: intensive care unit, Q1: first quartile, Q3: third quartile.

## Data Availability

The datasets used and/or analyzed during the current study are available from the corresponding author on reasonable request.

## Abbreviations

APACHE: Acute Physiologic Assessment and Chronic Health Evaluation
CCI: Corrected count increment
CI: Confidence interval
DIC: Disseminated intravascular coagulation
ICU: Intensive care unit
OR: Odds ratio
PI: Platelet increment
Q: Quartile.
